# A potential revolution in cancer treatment: A topical review of FLASH radiotherapy

**DOI:** 10.1002/acm2.13790

**Published:** 2022-09-27

**Authors:** Yuan Gao, Ruirui Liu, Chih‐Wei Chang, Serdar Charyyev, Jun Zhou, Jeffrey D. Bradley, Tian Liu, Xiaofeng Yang

**Affiliations:** ^1^ Department of Radiation Oncology and Winship Cancer Institute Emory University Atlanta Georgia USA

**Keywords:** cancer, FLASH, radiotherapy

## Abstract

FLASH radiotherapy (RT) is a novel technique in which the ultrahigh dose rate (UHDR) (≥40 Gy/s) is delivered to the entire treatment volume. Recent outcomes of in vivo studies show that the UHDR RT has the potential to spare normal tissue without sacrificing tumor control. There is a growing interest in the application of FLASH RT, and the ultrahigh dose irradiation delivery has been achieved by a few experimental and modified linear accelerators. The underlying mechanism of FLASH effect is yet to be fully understood, but the oxygen depletion in normal tissue providing extra protection during FLASH irradiation is a hypothesis that attracts most attention currently. Monte Carlo simulation is playing an important role in FLASH, enabling the understanding of its dosimetry calculations and hardware design. More advanced Monte Carlo simulation tools are under development to fulfill the challenge of reproducing the radiolysis and radiobiology processes in FLASH irradiation. FLASH RT may become one of standard treatment modalities for tumor treatment in the future. This paper presents the history and status of FLASH RT studies with a focus on FLASH irradiation delivery modalities, underlying mechanism of FLASH effect, in vivo and vitro experiments, and simulation studies. Existing challenges and prospects of this novel technique are discussed in this manuscript.

## 1 INTRODUCTION

Radiotherapy (RT) is a noninvasive treatment strategy to combat human tumors. According to some estimation, 50%–60% of cancer patients need RT alone or in combination with other treatment strategies.^1^ The primary treatment goal of RT is local control of tumor, as the ionizing radiation can directly or indirectly induce damage to tumor cells,^2^ while causing no or minimal side effect in normal tissue. The radiation oncology community has been working decades to improve the effect of killing tumor cells and minimizing the negative impact on normal cells at the same time. The best way to achieve this goal is to increase the therapeutic window by increasing the tumor control probability (TCP) over normal tissue complication probability (NTCP).^3^ In the past two decades, new advanced technologies have been developed, such as intensity‐modulated RT,^4^ stereotactic body RT,^5^ and pencil beam scanning (PBS) proton therapy.^6^ Recently, a sequence of research has shown that ultrahigh dose rate (UHDR) (>40 Gy/s) has a protective effect on normal tissue, which was first defined as “FLASH” effect by Favaudon et al. in 2014.^7^ They showed that the normal smooth muscle was spared when receiving an UHDR (>40 Gy/s, FLASH) compared to conventional (≤0.03 Gy/s, CONV) dose rate irradiation,^7^ and FLASH was as efficient as CONV in tumor growth control. This novel technique can lead to a higher TCP/NTCP.

**FIGURE 1:**
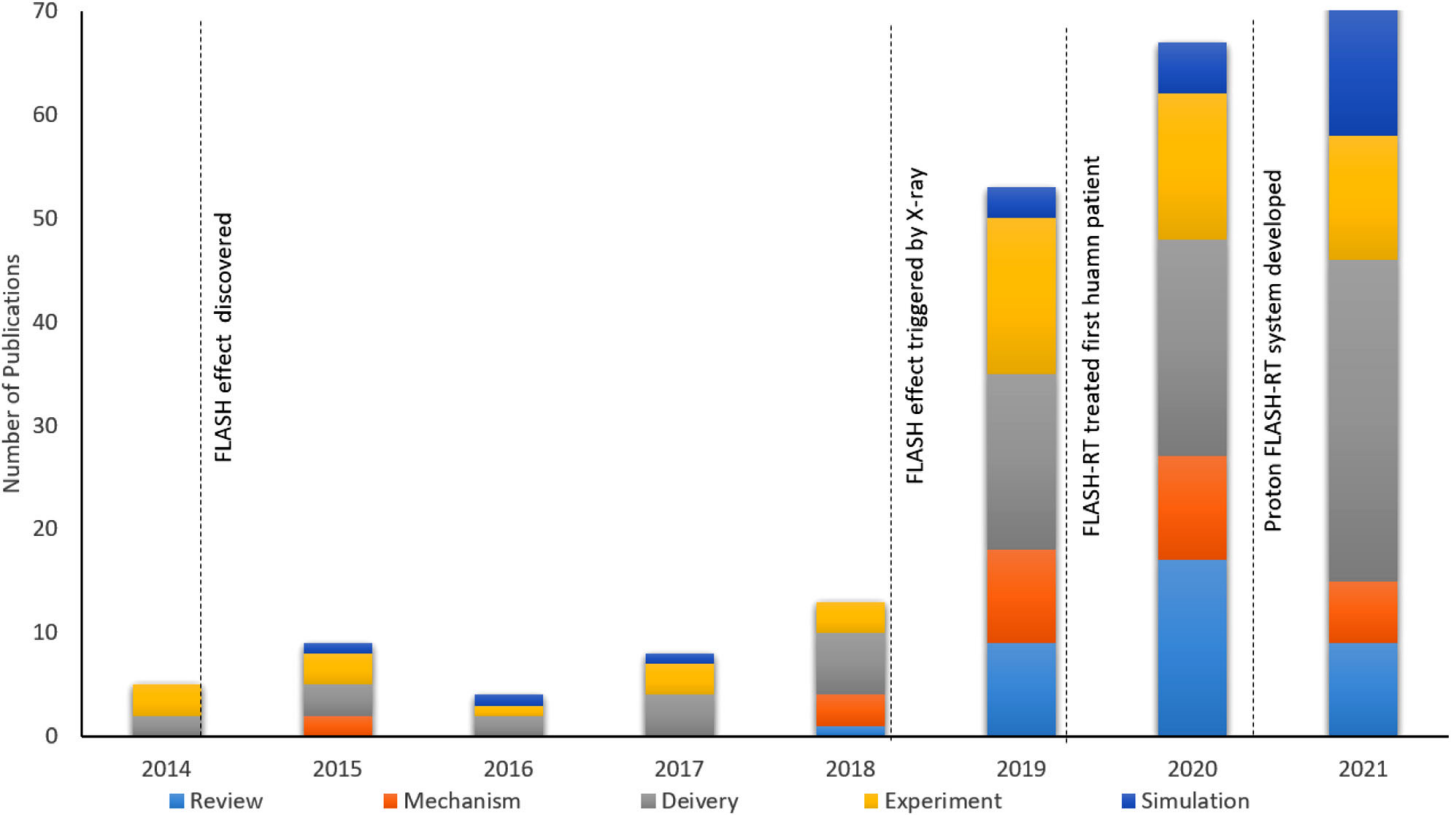
FLASH‐related publications since 2014: the 2021 publications were collected until October 2021.

There is a significant increase in FLASH research publications in recent years. We have collected the publications that include the keyword “FLASH RT,” “UHDR,” and the publication trend is shown in Figure 1. This manuscript is built on many existing UHDR research articles. We covered contemporary FLASH‐RT research (until October 2021) and included more related research fields (biology, physics, simulation, and experiments) rather than one or two specific fields.^8^, ^9^, ^10^, ^11^, ^12^ We want to provide the medical physics community with a general library to understand the current research progress in FLASH‐RT. The summarized information about publications is shown in Tables 1, 2, 3, 4, 5, 6, 7. Given the FLASH effect is not fully understood in many aspects, we hope this review article can assist researchers in guiding their scientific investigations.

**TABLE 1:** Selected publication that realized FLASH irradiation with electrons

Reference	Machine	Energy (MeV)	Dose rate (Gy/s)	Repetition rate (Hz)	Assay
Bazalova‐Carter, Liu^58^	NLCTA	50–70	$3\;\times \;10^{12}-9\;\times 10^{12}$	1	Hardware
Gamba, Corsini^61^	CLEAR	200	N/A	0.833–5	Hardware
Delorme, Marchand^62^	PRAE	30–70	N/A	50	Hardware
Felici, Barca^67^	NOVAC7	3/5/7/9	Up to 540	5–30	Hardware
Laschinsky, Karsch^68^	ELBE	20	10^5 ^(mean) 10^9^(max)	Quasi‐continuously	In vitro experiment
Kim, Gwak^45^	Varian 21 EX	9/20	352.1	100	In vitro experiment
Chabi, To^69^	Oriatron eRT6	6	200	100	In vitro experiment
Favaudon, Caplier^7^	Kinetron	4.5	60	19	In vivo experiment
Montay‐Gruel, Petersson^54^	Oriatron 6e	6	>100	100	In vivo experiment
Venkatesulu, Sharma^39^	Varian 2100 IX	20	37	N/A	In vivo experiment
Alaghband, Cheeks^57^	Oriatron eRT6	5.6	$4.4\;\times 10^{6}$	100	In vivo experiment
Pawelke, Brand^36^	ELBE	20	10^5 ^(mean) 10^9^(max)	Quasi‐continuously	In vivo experiment
Montay‐Gruel, Acharya^70^	Oriatron eRT6	5.6	Up to $7.8\;\times 10^{6}$	100	In vivo experiment
Bourhis, Sozzi^56^	Oriatron eRT6	5.6	150	100	Patient Treatment
Schuler, Trovati^63^	Varian 21 EX	9/20	74	180	Dosimetry
Jaccard, Duran^55^	Oriatron eRT6	6	Up to 200	200	Dosimetry
Favaudon, Lentz^53^	Kinetron	3.7–5.1	$2\;\times \;10^{2}-9\;\times 10^{7}$	1–250	Dosimetry
Lansonneur, Favaudon^52^	Kinetron LINAC	4.5	Up to $2\;\times \;10^{7}$	10–200	Dosimetry
Lempart, Blad^65^	ELEKTA	8	30–300	200	Dosimetry
Moeckli, Gonçalves Jorge^66^	Mobetron	6/9	700, 800	5–90	Dosimetry
Oesterle et al.^71^	Mobetron/Oriatron eRT6	6/12	N/A	30/100	Hardware
Konradsson et al.^72^	ELEKTA	8	30–300	200	Patient treatment

**TABLE 2:** Selected publications that realized FLASH irradiation with X‐rays

Reference	Machine/Facility	Energy (keV)	Mean dose rate (Gy/s)	Repetition rate (Hz)	Assay
Montay‐Gruel, Acharya^25^	ESRF	102	37	Continuous	In vitro experiment
Montay‐Gruel, Bouchet^73^	ESRF	102	37	Continuous	In vivo experiment
Smyth, Donoghue^74^	IMBL, Australia Synchrotron	95	Up to 319	Quasi‐continuous	In vivo experiment/dosimetry
Bazalova‐Carter and Esplen^75^	MXR‐160 MXR‐165	160	114/160	Continuous	Hardware
Kutsaev, Agustsson^77^	FLEX S‐band LINAC	9000	117.5	Continuous	Hardware
Rezaee, Iordachita^76^	RAD‐94	49.5	132/71.5	Continuous	Dosimetry

**TABLE 3:** Selected publications that realized FLASH irradiation with protons

Reference	Machine facility	Accelerator type	Energy (MeV)	Dose rate (Gy/s)	Delivery system	Assay
Auer, Hable^81^	SNAKE	Laser‐driven proton beam	20	≥10^9^ (pulse mode)	N/A	In vitro experiment
Buonanno, Grilj^41^	RARAF	Singletron	5.5	100 and 1000	Double scattering	In vitro experiment/dosimetry
Grilj, Buonanno^33^	RARAF	Singletron	5.5	100, 1000	Double scattering	In vitro experiment
Han, Mei^82^	CLAPA	Laser plasma accelerator	15	10^9^	Not provided	In vitro experiment
Yang, Lu^46^	CLAPA	Laser plasma accelerator	15	10^9^	Not provided	In vitro experiment
Zlobinskaya, Siebenwirth^83^	SNAKE	Laser‐driven proton beam	23	≥10^9^ (pulse mode)	N/A	In vivo experiment
Beyreuther, Brand^84^	UPTD proton beam	Not provided	224	100 (mean) 200 (0.5%)	PBS	In vivo experiment
Abel, Girdhani^85^	Not provided	Not provided	Not provided	40	PBS	In vivo experiment
Kourkafas, Bundesmann^86^	HZB	Cyclotron	68	75	Single scattering	In vivo experiment
Cunningham, McCauley^87^	Varian Probeam	Isochronous cyclotron	250	115.1	PBS	In vivo experiment/hardware
Patriarca, Fouillade^78^	IBA C230	Isochronous cyclotron	230	40 and 80	PBS	Hardware
Younkin, Bues^88^	Hitachi ProBeatV	Synchrotron	250	Not provided	PBS	Hardware
IBA^89^	IBA Proteus	Isochronous cyclotron	230	Up to 200	PBS	Hardware
Kolano^90^	AVO	LINAC	250	Not provided	PBS	Hardware
Darafsheh, Hao^91^	Mevion HYPERSCAN	Synchrocyclotron	230	100–200	Double scattering	Hardware/dosimetry
Nesteruk, Togno^80^	PSI Gantry 1	Cyclotron	250	1–9000	PBS	Hardware/dosimetry
Zou, Diffenderfer^92^	IBA	Cyclotron	226.2	160	Double scattering	Hardware/dosimetry
Diffenderfer, Verginadis^79^	IBA Proteus plus	Isochronous cyclotron	230	78	Double scattering	Dosimetry
Zhang, Cascio^32^	IBA C230	Isochronous cyclotron	227.5	120	Double scattering	Dosimetry
Kang et al.^93^	Varian Probeam	Isochronous cyclotron	250	115.1	PBS	Hardware/dosimetry

Abbreviation: PBS, pencil beam scanning.

**TABLE 4:** Selected MC simulation studies

Reference	Code	Assay
Bazalova‐Carter, Liu^58^	EGSnrc BEAMnrc/DOSXYZnrc	Dosimetry
Palma, Bazalova‐Carter^100^	DOSXYZnrc/EGSnrc	Dosimetry
Schuler, Trovati^63^	FLUKA	Dosimetry
Bazalova‐Carter and Esplen^75^	DOSXYZnrc/EGSnrc	Dosimetry
Lansonneur, Favaudon^52^	GATE 8.0 (Geant4)	Dosimetry
Darafsheh, Hao^91^	TOPAS	Dosimetry
Lagzda, Angal‐Kalinin^107^	TOPAS	Dosimetry/hardware
Simeonov, Weber^108^	FLUKA	Dosimetry/hardware
Rezaee, Iordachita^76^	Geant4	Dosimetry
Brunetti, Maitrallain^103^	Geant4	Dosimetry/radiation transport/hardware
Abolfath, Grosshans^109^	Geant4‐DNA	Molecular dynamics simulation/DNA simulation
Jay‐Gerin^110^	IONLYS‐IRT	Gamma ray electron‐radiolysis of water
Ramos‐Méndez, Dominguez‐Kondo^111^	TOPAS‐nBio	Radiochemistry/radiolysis simulation
Zakaria, Colangelo^112^	IONLYS‐IRT	Radiochemistry simulation
Alanazi, Meesungnoen^113^	IONLYS‐IRT	Radiolysis of water/oxygen consumption simulation
Tsai, Tian^114^	Geant4‐DNA, gMicroMC	DNA damage simulation
Lai, Tsai^115^	Geant4‐DNA, gMicroMC	DNA damage simulation
Lai, Jia^116^	Geant4‐DNA, gMicroMC	Radiolysis/DNA damage simulation
Lai, Jia^117^	Geant4‐DNA, gMicroMC	Radicals and DNA simulation
Mahbubur Rahman^118^	Geant4‐based GAMOS	Treatment plan
Small, Henthorn^119^	Geant4‐DNA	DNA damage simulation

**TABLE 5:** Summary of in vitro experiments investigating FLASH effect mechanism

Reference	Tumor and normal cells	Radiation source type	Total dose (Gy)	FLASH dose rate (Gy/s)	Results
Auer, Hable^81^	G2 phase cell, HeLa Cell	SNAKE 20 MeV Proton beam	3	≥10^9^ (Pulse mode)	The RBE of FLASH‐RT and CONV‐RT is not different significantly
Zlobinskaya, Siebenwirth^83^	FaDu	SNAKE 23 MeV Proton beam	1.4	≥10^9^ (Pulse mode)	No different in RBE with CONV‐RT
Laschinsky, Karsch^68^	One human cell line and two human primary fibroblasts	ELBE 20 MeV quasi‐continuous Electron beam	4, 8	10^5 ^(mean) 10^9^(max)	Proved the radiobiological effectiveness of the pulsed electron beams is not affected by FLASH‐RT
Buonanno, Grilj^41^	Normal human lung fibroblasts (IMR90)	4.5 MeV RARAF protons	20, 10	100, 1000	Proton FLASH‐RT mitigated detrimental effects
Fouillade, Curras‐Alonso^123^	Mice with human cells	4.5 MeV electron LINAC	5.2 and 4	>20	FLASH minimizes DNA damage in normal cells
Grilj, Buonanno^33^	IMR90, A549, HAP1 TSA	RARAF 5.5 MeV Proton beam	2–10	0.1, 10, 100	No dose‐rate‐dependent variation was observed between the survival fraction of cancer cells
Yang, Lu^46^	MCF‐7 cells, MCF‐7 CSCs.	CLAPA 15 MeV proton beam	6–9	10^9^	CSC is more resistant to radiation than normal cancer cell under FLASH‐RT, a potential mechanism
Kim, Gwak^45^	Mice injected with Lewis lung carcinoma cells	Varian 21 EX, Electron LINAC	15	352.1	MLC activation in tumors may be responsible for some of the tumor microenvironment change
Han, Mei^82^	Cyt *c*‐normal and ‐null mice embryonic fibroblast cells	CLAPA 15 MeV proton beam	30	10^9^	FLASH‐RT induced significant early apoptosis

Abbreviations: CSC, cancer stem cell; MLC, myosin light chain; RT, radiotherapy.

**TABLE 6:** Normal tissue sparing effect from FLASH‐radiotherapy (RT)

Reference	Model	Radiation source type	Total dose (Gy)	FLASH dose rate (Gy/s)	Results, FLASH effect (Yes/No)
Favaudon, Caplier^7^	Mice	Kinetron LINAC (electron)	17(25)/15(28)	60	Yes. FLASH‐RT protects lungs from radiation‐induced fibrosis and protects blood vessels and bronchi from acute apoptosis
Montay‐Gruel, Petersson^54^	Mice	Oriatron 6e (electron)	10	>100	Yes. The mice normal brain tissue toxicities reduced after FLASH‐RT
Loo, Schuler^129^	Mice	N/A (electron)	10–22	210	Yes. Significantly increased survival with FLASH‐RT
Montay‐Gruel, Bouchet^73^	Mice	ESRF (X‐ray)	10	37	Yes. FLASH‐RT does not induce memory deficit; reduce hippocampal cell‐division impairment, and less reactive astrogliosis
Simmons, Lartey^64^	Mice	Varian 21EX (electron)	30	200–300	Yes. Reduced cognitive deficits after FLASH‐RT
Abel, Girdhani^85^	Mice	N/A (proton)	15/17.5/20	40	Yes. Radiation‐induced skin toxicity is lowered with FLASH‐RT
Venkatesulu, Sharma^39^	Mice	Varian 2100 IX (electron)	16	37	No. The FLASH proton feasibility was shown, but the FLASH effect was not significant
Zhang, Cascio^32^	Mice	IBA C230, (proton)	13–22	120	Yes. FLASH‐RT is less harmful to the mice
Alaghband, Cheeks^57^	Mice	Oriatron eRT6 (electron)	8	$4.4\;\times 10^{6}$	Yes. FLASH‐RT was found to ameliorate radiation‐induced cognitive dysfunction. Normal tissue toxicities reduced
Fouillade, Curras‐Alonso^123^	Mice	N/A (electron)	5.2/4	>20	Yes. FLASH minimizes DNA damage in normal cells
Soto, Casey^128^	Mice	N/A (electron)	30/40	180 (average)$\;4\;\times 10^{5}$ (pulse)	Yes. FLASH‐RT results in both a lower incidence and severity of skin ulceration
Levy, Natarajan^130^	Mice	N/A (electron)	16	216	Yes. FLASH‐RT produced less mortality, spared cell death
Diffenderfer, Verginadis^79^	Mice	IBA Proteus Plus (proton)	12–18	78	Yes. FLASH‐RT at 15 Gy significantly reduced the loss of proliferating cells in crypts, and a reduction of intestinal fibrosis at 18 Gy
Allen, Acharya^125^	Mice	Oriatron eRT6 (electron)	10, 25	$5.6\;\times 10^{6}$ $2.5\;\times 10^{3}$	Yes. FLASH‐RT reduces levels of apoptosis of brain and minimized vascular dilation
Montay‐Gruel, Markarian^126^	Mice	Oriatron eRT6 (electron)	10	$5.6\;\times 10^{6}$ $2.5\;\times 10^{3}$	Yes. FLASH‐RT reduces reactive gliosis in mice brain
Montay‐Gruel, Acharya^70^	Mice	Oriatron eRT6 (electron)	10/14/25/30	10/14	Yes. FLASH‐RT was found to significantly spare radiation‐induced cognitive deficits
Cunningham, McCauley^87^	Mice	Varian Probeam (proton)	35	57	Yes. Skin and soft tissue toxicity was reduced with FLASH‐RT
Chabi, To^69^	Mice	Oriatron eRT6 (electron)	4	200	Yes. FLASH‐RT reduces functional damage to human blood stem cells
Velalopoulou, Karagounis^131^	Mice	IBA Proteus Plus (proton)	30/45	69–124	Yes. FLASH‐RT can spare murine skin, muscle, and bone
Ruan et al.^127^	Mice	6‐MeV electron linear accelerator	7.5–12.5	>280	Yes. Higher the average dose rate, the larger the FLASH effect
Smyth, Donoghue^74^	Mice	IMBL (X‐ray)	12.7–587	44.4	No. No evidence of a normal tissue sparing effect
Montay‐Gruel, Acharya^25^	Mice/zebrafish	ESRF (X‐ray)	10/14	37	Yes. FLASH‐RT did not cause radiation‐induced deficits in learning and memory in mice
Beyreuther, Brand^84^	Zebrafish embryos	UPTD (proton)	0–45	100	No. The FLASH proton feasibility was shown, but the FLASH effect was not significance
Pawelke, Brand^36^	Zebrafish embryos	ELBE (electron)	26	10^5 ^ (mean) 10^9^ (max)	Yes. FLASH effect was seen for most endpoints
Vozenin, De Fornel^99^	Pig skin	Kinetron (electron)/Oriatron 6e (electron)	22–34	300	Yes. The skin toxicity is smaller after FLASH‐RT
Buonanno, Grilj^41^	Normal human lung fibroblasts (IMR90)	RARAF (proton)	20/10	100/1000	Yes. Mitigated long‐term detrimental effects senescence

**TABLE 7:** Summary of publications focusing on tumor control with FLASH‐radiotherapy (RT)

Reference	Model	Radiation source type	Total dose (Gy)	FLASH dose rate (Gy/s)	Outcome
Favaudon, Caplier^7^	Mice, HBCx‐12A and Hep‐2 human xenografts; mice, orthotopic tumor model comprising TC‐1 cells	Kinetron LINAC (electron)	17(25)/15(28)	60	FLASH‐RT is as efficient as CONV‐RT in controlling xenografted human tumors and orthotopic lung tumors
Zlobinskaya, Siebenwirth^83^	NMRI mice with FaDu cells	SNAKE (proton)	17.4 and 19.7	≥10^9^ (Pulse mode)	No difference in RBE and tumor growth delay, induced by FLASH‐RT and CONV‐RT
Rama, Saha^44^	C57BI/6J mice with	(proton)	18	40	FLASH‐RT induced more efficient lung‐tumor eradication than CONV‐RT
Vozenin, De Fornel^99^	Cat, T2/T3N0M0 squamous‐cell‐carcinoma	Kinetron (electron) Oriatron 6e (electron)	25–41	130–190	Tumor growth is under control after single‐dose FLASH‐RT
Bourhis, Sozzi^56^	Human, CD30+ T‐cell cutaneous lymphoma	Oriatron eRT6 (electron)	15	167	Tumor response was rapid complete and durable within 5 months
Diffenderfer, Verginadis^79^	Mice, pancreatic cancer flank tumors	IBA Proteus Plus (proton)	15/12–18	78	FLASH‐RT and CONV‐RT tumor growth inhibition is preserved
Levy, Natarajan^130^	Mice, total abdominal irradiation	N/A (electron)	16	216	The FLASH‐RT has similar efficacy in reducing tumor burden
Cunningham, McCauley^87^	Mice, with MOC1 and MOC2 head and neck cancer	Varian Probeam (proton)	15	115	The tumor efficacy is similar with CONV‐RT
Montay‐Gruel, Acharya^70^	Mice injected with glioblastoma cells	Oriatron eRT6 (electron)	25	$2.5\;\times 10^{3}$ to $7.8\;\times 10^{6}$	FLASH‐RT are same with CONV‐RT in delaying glioblastoma growth
Chabi, To^69^	Mice. Total body irradiation, on humanized model of T‐ALL	OriatroneRT6 (electron)	4	200	FLASH‐RT has a therapeutic effect on human T‐ALL with common profile

## 2 THE MECHANISM FOR FLASH EFFECT

The FLASH effect is defined as the reduction of radiation‐induced damage in normal tissue under UHDR irradiation.^12^ To date, the underlying mechanism for the FLASH effect has not been fully understood, and its investigation turns to be a hot topic in radiation oncology community. Currently, the most popular hypotheses on the FLASH effect mechanism are (a) oxygen depletion and reactive oxygen species (ROS), (b) immune and inflammatory processes.

### 2.1 Oxygen depletion

Oxygen depletion hypothesis suggests that the rapid oxygen depletion in normal tissue under FLASH irradiation renders the normal tissue radioresistant to the radiation.^13^ The relationship between radiation dose rate and oxygen consumption was revealed in 1959 with a bacteria study,^14^ and the experiment shows that the bacteria have a higher survival rate with a higher delivered dose rate, which might be because of the bacteria in hypoxic state. The reason that the hypoxic tissues are more radioresistant than normal‐oxygenated tissue has been fully investigated.^15^, ^16^, ^17^ For the low linear energy transfer (LET) radiation, the DNA damage results from ROS generation that can induce damage to DNA.^2^, ^18^, ^19^, ^20^ A cell in a hypoxic environment can have more radioresistance than that in a normal oxygen environment. Labarbe et al. developed a physicochemical model of reaction kinetics to investigate the peroxyl radical generation impact on FLASH effect. Their model showed that the shortened radical recombination under FLASH‐RT can shorten or limit the radiolytic yield of peroxyl radical, which can protect the normoxic tissue against radiation induced damage.^21^ The detailed review on the investigation of hypoxic tissue radioresistance to radiation, DNA response, and repair was performed in 2008.^15^ The oxygen depletion hypothesis suggests that the local oxygen depletion process is faster than the reoxygenation process during the FLASH‐RT, and thus the normal tissue is under hypoxia condition. Therefore, the normal tissue is more radioresistant under FLASH irradiation.^18^, ^22^, ^23^, ^24^ The oxygen depletion hypothesis provides an opportunity to study the mechanism through vitro and vivo experiments. This hypothesis was directly tested on mouse brain by a group of researchers in Switzerland.^25^ They concluded that FLASH‐RT can reduce the production of ROS and H_2_O_2_, which can lead to the reduction of the radiation‐induced DNA damage to normal tissues. Vozenin et al. pointed out that FLASH irradiation experiments on the aerobic cells (21% oxygen) did not reveal FLASH effect because of the high oxygen tension, making the oxygen depletion insufficient.^26^ It is important to design in vitro experiments under normal tissue oxygen levels, which vary from 4% to 7.5% with an average of 5%.^24^ Two models of oxygen depletion during FLASH‐RT were developed by Pratx's group, and predications were made that the FLASH effect might be only observed in the hypoxic cell. Given this, the change of oxygen tension might reduce FLASH effect.^23^ Pratx et al. implanted physiobiological equations into models to investigate FLASH effect within hypoxic multicellular tumor spheroids through simulation and experiments. The improved survival of tumor spheroids under FLASH‐RT confirms the oxygen depletion.^27^ In order to better understand the contribution of oxygen to the FLASH effect, Pratx et al. developed a 3D computation model to measure oxygen in vivo during FLASH‐RT. They concluded that the process on the order of milliseconds is recommended for radiochemical oxygen depletion measurements in normal tissues.^28^ Rothwell et al. presented their modeling work about oxygen depletion by implanted biological, radiochemical, and delivery parameters.^29^ Contrary to the oxygen depletion models raised by Pratx et al.,^23^ they used effective diffusivity to account for the porous nature of space between cells. Their model provided a framework for further investigation and experiment design for FLASH effect, and the initial results support the experimental evidence. Petersson et al. conducted a quantitative study on the oxygen tension during FLASH‐RT, and their model reproduced the oxygen tension dependence of normal tissue responses to FLASH‐RT.^30^ A recent study on FLASH effect on oxygen concentration shows the evidence of oxygen depletion, causing the normal tissues’ different response to CONV‐RT and FLASH‐RT.^31^ The FLASH effect on normal tissues was confirmed with a proton beam due to the oxygen depletion mechanism.^32^ The generation of ROS during FLASH‐RT also contributes to the FLASH effect, because of its different biochemistry process between normal tissues and tumors.^12^ An in vivo experiment irradiating the zebrafish embryos using conventional dose rate and FLASH dose rate provided the evidence that FLASH‐RT makes the normal tissue more radioresistant by a reduced production of ROS.^25^ The oxygen depletion hypothesis and ROS reduction might be able to explain the reduced DNA damage of normal tissues with FLASH‐RT, but why the tumor maintains the same response to CONV‐RT has not been fully investigated.^8^ A possible explanation is presented in recent publication. The higher levels of redox‐active iron in tumor and the different oxidative metabolism in normal tissues and tumor might be the determinant of the tumor maintaining the response.^18^ A water radiolysis study published recently revealed that the oxygen dissolved in water is not completely depleted with proton dose as low as 10 or 20 Gy at a homogeneous dose rate of 1000 Gy/s. On the other hand, their data show that the oxygen can be fully depleted at proton doses of 107 and 56 Gy at 1000 Gy/s for samples with 21% and 4% oxygen.^33^ However, a recent experimental study found that the FLASH‐RT does consume oxygen, but not enough to deplete all the oxygen, and oxygen hypothesis is not a suitable mechanism to explain the FLASH effect alone.^34^ A quantification measurement of oxygen depletion during FLASH‐RT in vitro and in vivo is published recently. They reported that the oxygen depletion to radiologically relevant hypoxia is unlikely to occur in bulk tissues under FLASH‐RT, whereas the oxygen depletion comparison between FLASH and conventional irradiation in vivo can be quantified, due to the resupply of oxygen from blood.^35^ The higher dose rate study on this still needs to be conducted to compare with previous contradicting result. Pawelke et al. presented an oxygen depletion experiment by considering the partial oxygen pressure as a relevant parameter.^36^ They confirmed that a protective FLASH effect was observed at specific partial oxygen pressure in zebrafish embryos. Favaudon et al. reviewed the model study on the role of oxygen in the FLASH effect, and they focused on the observations supporting or refuting three models studies that include oxygen depletion, ROS, and self‐annihilation of radicals.^37^

### 2.2 Immune and inflammatory responses

Except the oxygen depletion hypothesis, the immune and inflammatory responses have also been proposed as the mechanism that contributes to the FLASH effect. FLASH‐RT may have a direct or indirect impact on immune cells and tumor microenvironment.^38^ The FLASH effect is not found in FLASH irradiation experiment on immune cells.^39^ TGF‐β (transforming growth factor beta) is an important pro‐inflammatory cytokine, a major regulator of antitumor immunity followed by FLASH‐RT.^12^, ^40^ One study on the biological effect of normal cells under FLASH‐RT has proposed that the TGF‐β might contribute to the FLASH effect.^41^ The discussion and investigation about TGF‐β and T‐cell that resist radiation continue.^40^, ^42^, ^43^ Rama et al. performed an FLASH‐RT experiment using a clinical PBS proton system, which shows that FLASH‐RT induced more efficient lung‐tumor eradication and improved the recruitment of T lymphocytes compared to CONV‐RT.^44^ This provides more evidence for the immune response hypothesis. The CD&αT cell influx in tumors increased by FLASH‐RT was also observed in a recently published study.^45^

### 2.3 Other potential hypotheses

Both oxygen depletion and immune response hypothesis need a lot of efforts to be verified through theory and modeling developments in radiochemistry, biochemistry, and physics, as well as with in vivo and in vitro experiments. Investigating the underlying mechanism of FLASH effect until fully understood is important for the translation of FLASH‐RT to clinic. A group of scientists reported for the first time the killing effects and death pathways of cancer stem cells (CSCs) and normal cancer cells under FLASH‐RT, and this work might help the community to further understand the CSC radio‐resistance.^46^ A study published recently shows that myosin light chain activation in cancer cells and tumor vasculature may contribute to the FLASH effect.^45^ Another computational study performed directly models the effect of radiation dose rate on the killing of circulating immune cells. They reported a strong sparing effect on circulating immune cells by FLASH‐RT, suggesting that this might contribute to the FLASH effect.^47^

### 2.4 Discussion

The FLASH irradiation is delivered in several nanoseconds much shorter than CONV‐RT; the recent experiments revealed that the hypoxic normal tissues cannot get reoxygenation process performed in such a short time period, then the normal tissues get protected against radiation.^48^ Hypoxic normal tissues in such a short period (few nanoseconds) cannot be detected by a response of hypoxia‐mediated markers.^20^ More efforts on how to track the oxygen level changes during FLASH‐RT are needed. The radiomics studies to prove (disprove) the oxygen depletion hypothesis are still to be performed. The oxygen depletion might explain why the FLASH‐RT can spare normal tissues, but it still cannot explain why FLASH‐RT has the similar tumor control ability with CONV‐RT. Zhou et al. proposed that the significant difference in oxygen level between normal tissues and tumors might explain this,^49^ which needs more experimental proofs. Although the FLASH effect has been observed in vitro experiments, it is still unclear whether immune response to the FLASH‐RT contributes to the FLASH effect. More work is needed to clarify if the immune and inflammation response is different under FLASH‐RT and CONV‐RT and to find out if they are the underlying mechanism of FLASH effect.

## 3 DELIVERY MODALITIES

The research on UHDR delivery modalities is one of the most important steps in this emerging technology. Currently, UHDR delivery modalities can be categorized into three types according to the radiation source: electron, proton, and photon (X‐ray). The first inspiring research for FLASH RT purposes was conducted by the Favaudon and Vozenin group in Lausanne and Orsay, and the differential effect between tumor and normal tissues under FLASH‐IR was discovered in their seminal work.^7^ The FLASH irradiation was conducted with a 4.5‐MeV electron linear accelerator (LINAC), whereas the CONV irradiation was performed with the same machine but with a lower cathode current. A lot of research on delivering FLASH has been performed recently, after the seminal approach by Favaudon et al.^7^ Several researchers summarized and reviewed that research in recent publications. Wilson et al. categorized the existing irradiation delivery modalities into electron, proton, X‐ray and made a comparison among those techniques in detail in their review article.^20^ Breitkreutz et al. reviewed the history and status of kilovoltage X‐rays application in CONV‐RT, and they discussed the X‐ray‐delivered modalities in FLASH‐RT.^50^ Esplen et al. did a detailed review of radiation sources for FLASH RT. They discussed three kinds of radiation sources and summarized the current capable and prospective delivery modalities.^51^ They performed a detailed review of the dosimetry problems in FLASH‐RT, which is not included in this work. Jolly et al. summarized the current FLASH RT delivery modality with proton and categorized the current commercial proton therapy system into four types according to their radiation source type.^10^ We summarized the delivery modalities according to their particle types, electron, X‐ray photon, and proton. We covered the most recent delivery modality approaches in recent publications.

### 3.1 Electrons

#### 3.1.1 Experiment electron accelerator

The first preclinical FLASH RT was performed by Favaudon et al. with a Kinetron LINAC in 2014, which emits 4.5‐MeV electrons.^7^ The average dose rate was about 60 Gy/s with dose per pulse of $5\;\times 10^{6}$ Gy. The LINAC was used to conduct a mouse FLASH study. Lansonneur et al. confirmed the Kinetron capability on UHDR delivery, and they concluded that such delivery modality can be adapted to conduct FLASH‐RT preclinical research experiments.^52^, ^53^

To date, two centers, Marie Curie Institute and University of Lausanne, lead the FLASH‐RT research using Kinetron and Oriatron to deliver UHDR irradiation. These LINACs can deliver electron beams at an average dose rate from 0.1 to 1000 Gy/s.^26^, ^54^ The Oriatron eRT6 developed by PMB Alcen is an experimental UHDR LINAC, which was designed to deliver an electron beam with variable dose rates ranging from 0.01 to over 100 Gy/s.^55^ Jaccard et al. have performed the commissioning and beam monitoring of the Oriatron eRT6 prototype.^55^ The first FLASH‐RT human patient was treated with Oriatron eRT6 LINAC in 2019. This experimental facility can deliver 5.6‐MeV high‐energy electron beams.^56^ Oriatron eRT6 was deployed in another study to irradiate the entire brain of juvenile mice, and the highest delivered dose rate was $4.4\;\times \;10^{6}$ Gy/s.^57^

The NLCTA (Next Linear Collider Test Accelerator) is an experimental LINAC developed by SLAC (SLAC National Accelerator Laboratory), and it was employed to investigate the use of very high‐energy electrons (VHEE) for FLASH RT.^58^ The NLCTA beam can deliver 50–70‐MeV VHEE beams, and the measured dose rate can be up to $9.0\;\times \;10^{12}$ Gy/s with 60‐MeV electron beams.^58^ After proving the ability of NLCTA for UHDR delivery, Bazalova‐Carter et al. moved forward and developed a treatment planning workflow for FLASH RT with VHEE pencil beams.^59^ To date, there is no clinical facility available for VHEE treatment; however, a perspective on medical treatment platform, pluridirectional high‐energy agile scanning electron RT (PHASER), is being developed at the SLAC.^60^ The PHASER still needs to overcome challenges from the clinical and technological point of view. The increasing interest in FLASH RT stimulates scientists to deploy VHEE beam for clinical treatment. The CERN Linear Electron Accelerator for Research (CLEAR) facility was approved in December 2016, and it focuses on the future accelerator applications, including VHEE capability on FLASH RT.^61^ The Platform for Research and Applications with Electrons (PRAE) facility is under construction in France, which will deliver a pulsed electron beam in the energy range 30–70 MeV.^62^ A superconducting linear electron accelerator called ELBE (Electron Linac for beams with high Brilliance and low Emittance) can deliver 20‐MeV quasi‐continuous (13 MHz) electron beam, and the delivered beam dose rate can be up to 10^9^ Gy/s with the mean dose rate of 10^5^ Gy/s.^36^

#### 3.1.2 Clinical LINAC

There is a possibility that the first VHEE clinical facility will not be available in the next decade.^51^ Several clinical LINACs have been modified to deliver FLASH dose rate. A Varian Clinac 21 EX was modified to investigate the electron beam FLASH RT capability, and this modified LINAC can deliver electron beam with average dose rate ranges from 35 to 210 Gy/s. Kim et al. removed the treatment head cover of Varian 21 EX in the FLASH mode, the jaws of which were fully opened and an electron beam with a dose rate up to 352.2 Gy/s (instantaneous dose rate) was delivered.^45^ A spare 20‐MeV program printed circuit board was used to tune the LINAC system in this work, which can control parameters that include pulse forming network voltage, injector current, dosimetry calibration, and beam steering.^63^ A gantry head of a decommissioned Varian 2100 IX LINAC was disassembled and modified to deliver 20‐MeV electrons at a 37‐Gy/s dose rate.^39^ The Varian 21 EX's FLASH dose rate delivery capability was confirmed in another in vivo study.^64^ An ELEKTA Precise was modified to investigate if the UHDR beam can be generated by a clinical LINAC.^65^ A group of engineers modified the Mobetron to deliver FLASH beam by adjusting the delivery beam parameters, and the FLASH version of Mobetron is now being installed at multiple institutes by IntraOp (California, USA). The modified system can deliver up to 800 Gy/s (instantaneous dose rate) dose rate with 9‐MeV electron beam.^66^ Table 1 lists the summary of publications that successfully delivered FLASH dose rate with electron beams. Important parameters for electron beam control are listed in that table.

### 3.2 X‐rays

To date, only few studies proved the FLASH effect with X‐ray sources. Montay‐Gruel et al. first proved that the FLASH effect can be triggered by X‐ray beam. The FLASH‐RT was performed at the ID17 Biomedical Beamline of the ESRF (Grenoble, France), and 10 Gy was delivered to the entire brain of a mouse in a whole‐brain irradiation (WBI) with a synchrotron accelerator.^73^ An Australian group performed FLASH irradiation on mice with microbeam radiation therapy and synchrotron broad beam radiation therapy at IMBL (Imaging and Medical Beamline) of the Australian Synchrotron.^74^ Two conventional X‐ray tubes were modified to deliver FLASH‐RT, and the dose rate measurement results showed the capabilities of the modified conventional X‐ray tubes to deliver UHDR.^75^ A self‐shielded kilovoltage (kV) X‐ray cabinet was proposed with MC simulation to perform that the FLASH irradiation can be achieved by changing the position of X‐ray sources and anodes.^76^ Unfortunately, kV X‐rays are not fully suited for the treatment of deep‐seated tumors,^51^ so the development of megavoltage (MV) X‐rays has attracted more attention. The first MV X‐ray experimental platform was developed based on ARIEL e‐LINAC at TRIUMF. The modified e‐LINAC can deliver 10‐MeV electron beam, and the high‐energy electron beam can be used to produce a 10‐MV X‐ray beam.^51^ RadiaBeam Technologies (California, USA) has presented an S‐band accelerator that can deliver high‐energy X‐ray, named flexible LINAC for electrons and X‐rays (FLEX). Being initially designed for adaptive cargo inspection, this system has a great perspective on FLASH RT.^77^ Table 2 lists the summary of publications that successfully delivered FLASH irradiation with X‐rays.

### 3.3 Protons

The proton FLASH‐RT has also attracted a lot of interest from the radiation oncology community. The FLASH effect with protons was shown in a recent publication by Buonanno et al. who used a proton FLASH irradiator at the Radiological Research Accelerator Facility (RARAF). That facility can produce a pulse‐mode proton, accelerated by Singletron accelerator, with a dose rate range from 0.025 to 1500 Gy/s.^41^ However, many technical challenges with translation of conventional proton beam to FLASH proton beam remain. Several experimental setups have been performed with proton FLASH delivery.^78^, ^79^, ^80^ There are three major discussions in the progress of adapting the conventional proton beam to FLASH RT: the clinical dose rate requirement, the accelerator type, and the FLASH dose delivery system. The clinical dose rate requirements have not been established. Jolly et al. performed a detailed discussion on the quantitative requirements, including several important parameters in their review paper recently.^10^ The novel change in adapting conventional proton beam accelerator to FLASH irradiation is discussed according to the accelerator type and UHDR delivery system. Three kinds of clinical proton beam systems that can deliver FLASH irradiations: cyclotron, synchrotron, synchro‐cyclotron,^10^ and a potential proton therapy system LINAC proton beam. Table 3 lists a summary of publications that successfully delivered FLASH with proton beams.

#### 3.3.1 Cyclotron

The most common proton accelerator type–realized FLASH delivery is the cyclotron.^10^ C230, a clinical cyclotron‐based proton facility manufactured by IBA (Ion Beam Application), can be modified to deliver UHDR irradiation for small animal FLASH experiments. The UHDR was obtained after a single scattering system design for a 12‐mm field size.^78^ The capabilities of C230 to deliver FLASH irradiations have been verified by several studies.^32^, ^79^, ^92^ The ProBeam manufactured by Varian has also shown the capabilities to deliver FLASH irradiation.^94^ The ProBeam system is based on an isochronous cyclotron, which can accelerate protons with energies up to 250 MeV. A FLASH effect experiment on a single eye of mice was performed with an experimental proton beam facility, Helmholtz‐Zentrum Berlin für Materialien und Energie (HZB), which is also based on a cyclotron accelerator.^86^

#### 3.3.2 Synchrotron

The proton beam facility with a synchrotron accelerator is not as common as cyclotron‐based facility because of the increased complexity. There are more challenges to deploying the synchrotron‐based proton therapy facility to deliver FLASH irradiation than that to cyclotron‐based facility, especially regarding significant changes that need to be made on the design and operation.^10^ A group of scientists demonstrated that the synchrotron‐based proton RT facility can deliver FLASH irradiation with a modified ProBeatV medical system, which was manufactured by Hitachi.^88^, ^95^

#### 3.3.3 Synchro‐cyclotron

Synchro‐cyclotrons were introduced to the proton therapy community recently. They use a higher magnetic field and provide a smaller footprint. Proteus One, another clinical proton RT treatment system manufactured by IBA, uses synchro‐cyclotron to accelerate protons up to energies of 230 MeV. The IBA group has announced that they have successfully performed the FLASH irradiation with Proteus.^89^ Diffenderfer et al. used this system to deliver FLASH‐RT in their in vivo experiments.^79^ HYPERSCAN, a Mevion (Mevion Medical Systems, Littleton, MA) proton RT system based on the synchro‐cyclotron design, was modified to deliver FLASH proton irradiation for potential experiment. The modified machine can deliver 100 and 200 Gy/s average dose rate to a small field.^91^ RARAF is a proton irradiation facility at Columbia University (New York, NY) which contains two proton irradiation platforms: track‐segment irradiator and FLASH irradiator. Using a 5.5‐MeV Singletron accelerator, both can deliver the proton beam with dose rate up to 1000 Gy/s.^33^, ^41^

#### 3.3.4 LINAC

To date, there is no operational LINAC‐based proton RT treatment system worldwide. However, the LINAC‐based proton RT treatment system has a higher peak current, smaller beam emittance, and the ability to vary the energy pulse by pulse.^10^ LIGHT (LINAC for Image‐Guided Hadron Therapy) system, developed by AVO‐ADAM (Advanced Oncotherapy, Meyrin, Switzerland), aims for next‐generation proton RT treatment facility with a small and modular design, and based on a compact LINAC. The capabilities of LIGHT system to deliver FLASH irradiation have been performed by the scientists at AVO‐ADAM.^90^ Another system, compact laser plasma accelerator system (CLARA), was modified to irradiate CSCs with as high as 10^9^‐Gy/s proton beam in quest for a new mechanism of FLASH effect.^46^

#### 3.3.5 Proton beam delivery system

There are three major FLASH proton beam delivery system designs: double scattering, spot scanning, and hybrid system. Double‐scattering is the simplest way to meet the FLASH proton dose delivery requirement, and it has been realized by several groups.^32^, ^79^ As a more traditional arrangement, double‐scattering system has several limitations with its passive scattering design. In the double‐scattering system, the collimators and range shifters must be specific for each patient, and the dose is not very conformal to the treatment field, and there is a significant increase in neutron dose to the patient because of proton interaction with extra materials.^10^ The passive scattering (singe/double scattered) delivery system shows that it could be a candidate for FLASH‐RT owing to its much shorter irradiation time compared with spot scanning delivery system.^96^ Spot scanning system is more popular in the proton RT treatment, as the beam employed by such a system can be controlled in terms of position and intensity. The spot scanning arrangement has been performed to deliver FLASH proton irradiation in recent studies.^80^, ^97^ Spot scanning system has gained quite a lot attention from either big vendors or academic centers for achieving proton FLASH. Many research studies have been performed to achieve conformal FLASH using spot scanning machine.^98^ The third possible arrangement is the hybrid systems, a combination of double‐scattering and spot scanning systems.^10^ To date, there is no published study with hybrid systems.

### 3.4 Discussion

External beam RT delivery at UHDR for deep‐seated targets is one of the major challenges in translating FLASH‐RT into the clinic. We have summarized the successful delivered UHDR studies in this manuscript. To date, most FLASH experiments were performed with experimental low‐energy electron accelerators. The Kinetron and Oriatron eRT6 experimental electron LINACs were used at Marie Curie Institute and University of Lausanne, respectively, to perform FLASH studies on animal models and the first human patient.^7^, ^56^, ^99^ Experimental and clinical electron accelerators have shown their advantage in reliability, low cost, and potential to deliver FLASH dose rate. The difference in capabilities of accelerators still needs to be considered carefully when translating into clinical application.^51^ The clinical RT proton beam sources have been recognized as the potential platform for FLASH‐RT. The current operating clinical proton beam sources can be used to deliver FLASH dose rate with minor or even no modification.^79^ The most important limitation with current proton beam sources is the irradiated volumes that are very small, and a significant development is required in the future to overcome this limitation. Besides the limitations and challenges with each kind of delivery modality, general challenges for FLASH delivery techniques exist. The CONV‐RT requires five to seven intensity‐modulated beams. There is no such FLASH‐RT delivery system.^48^ The faster intensity modulation needs to be developed, as FLASH‐RT has a shorter irradiation time compared to CONV‐RT. As it is imperative to verify the beam delivery, the real‐time FLASH‐RT guidance system needs to be invented, as the FLASH‐RT delivered in shorter time and higher dose rate, and it required more precise motion management. The radiobiologic differences in FLASH‐RT between electron, photon, and proton sources need to be further investigated.

## 4 SIMULATION STUDY IN FLASH RESEARCH

### 4.1 Monte Carlo simulation studies

Simulation study is a good imitation of a real‐world experiment and could play an important role in FLASH effect research, especially in the new hardware development, dosimetry calculation, radiation‐induced damage on DNA, and radiolysis of water and free radical. The selected FLASH‐related simulation study publications are shown in Table 4. The most common use of MC is for the dose calculation. Bazalova‐Carter et al. used MC method to calculate the percentage depth dose for various beam sizes at 50 and 70‐MeV electron beams.^58^ This simulation was performed to make a comparison with a radiation dose measurement in a water‐equivalent material from a VHEE beams. The EGSnrc/BEAMnrc and DOSXYZnrc were used in this work to calculate dose in the polystyrene phantom. Even though the simulation showed good agreement (within 5%) with measurement data of depth–dose curves and beam profiles, it resulted in a 42% difference with measurement data when it comes to dose calculation. This result indicates that more investigation needs to be performed to fully understand the physics of VHEE beam interaction with matter alongside improving the accuracy of dosimetry devices. Palma et al. performed a VHEE beam dose distribution calculation for five clinical cases with the same MC code.^100^ The same MC code was used to model two 160 kV X‐ray tubes to perform dose calculation. The difference between simulation and experimental results was within 3.6%.^75^ The FLUKA code is a general‐purpose MC code for radiation transport and interaction with matter, which includes hadrons, heavy ions, and electromagnetic particles from few keVs to cosmic ray energies in materials.^101^ Schüler et al. performed a detailed dosimetry characterization with FLUKA and compared with experimental data.^63^ Geant4 is a toolkit for simulating the transport of particles in matter,^102^ which has developed features like particle tracing, geometry, and physics models. Throughout years, Geant4 has developed many features that can help researchers, including dose calculation, radiobiology (Geant4‐DNA), and many other extensions. Geant4 was used to perform dose calculation and new hardware design in FLASH research work.^76^, ^103^ Although GATE is one of the Geant4 applications for tomographic emission simulation purposes, it was used to model the dose distribution of a prototype electron beam LINAC to calculate the dose delivered to small animals.^104^, ^105^ The Gate8.0 (Geant4 4.10.3) was used to model the dose distribution of the beam and treatment head accelerator along the beamline. TOPAS (tool for particle simulation) is another MC simulation platform based on the well‐established MC code Geant4.^106^ TOPAS is widely used in medical physics field because of its user‐friendly feature. Darafsheh et al. used TOPAS to perform dose calculation of a FLASH proton irradiation experiment and compared with the integral depth–dose measurement data.^91^ TOPAS is also used in the VHEE beam design for FLASH irradiation and dose calculation work.^107^

Geant4‐DNA was developed based on and fully included in the general‐purpose Geant4 MC simulation toolkit with a focus on simulating biological damages induced by ionizing radiation at the cellular and subcellular scale.^120^ The FLASH effect mechanism is still not fully understood, and the Geant4‐DNA can help to investigate the hypothesis by performing the radiobiology simulation. Abolfath et al. presented the first‐principles molecular dynamics (MDs) simulation to investigate the oxygen depletion hypothesis.^109^ They used Geant4‐DNA to simulate the radiation damage to a segment of DNA in a box filled with H_2_O and O_2_ molecules. The Car–Parrinello MDs simulation was performed to calculate the rate through which H_2_O and O_2_ molecules convert to ROS. They showed that the oxygen depletion progress takes place within nanoseconds after FLASH irradiation, which is the most promising hypothesis to explain FLASH effect on normal tissues. The MD simulation is necessary to study the effect of radiation on formation and evolution of ROS over time. Abolfath et al. indicated that they are developing an interactive Geant4‐DNA‐MD platform to make such simulation work much easier. Small et al. evaluated the VHEE RBE from nanodosimetric pBR322 plasmid DNA damage.^119^ Geant4‐DNA was used in this work to simulate the radiation‐induced DNA damage, and the results were compared to experimental double stand break yields. TOPAS‐nBio, an extension of TOPAS, was developed in 2018 with a focus to advance the understanding of radiological effects at the subcellular scale.^121^ TOPAS‐nBio includes very low‐energy interactions of particles down to vibrational energies and can simulate particle interaction and propagates radiolysis products. TOPAS‐nBio can be used to simulate radiological experiments on cells by simulating the initial radiation‐induced damage and links to models of DNA repair kinetics. TOPAS‐nBio can be used to investigate the FLASH effect mechanism because of the features listed before. Ramos‐Méndez et al. presented their work on the development of TOPAS‐nBio.^111^ They added a new feature to TOPAS‐nBio allowing it to simulate inter‐track effects in the chemical stage of water radiolysis. They calculated the LET‐dependent *G* values of protons delivered in single pulse range from 1 ns to 10 μs with TOPAS‐nBio and compared with simulations done by no inter‐track setup. They found that the inter‐track reactions should be considered when investigating the FLASH‐RT‐induced biological damage. The newly developed feature in TOPAS‐nBio can assist the future studies in understanding FLASH effect such as exploring the radiation‐induced DNA damages in FLASH irradiation. A group at University of Texas Southwestern Medical center has developed a GPU‐based microscopic MC tool called gMicroMC to improve the computation efficiency of microscopic MC simulations.^114^, ^115^ The gMicroMC initially focused on the simulation for radicals produced from water radiolysis and now they have extended the feature to physical track simulation for energetic electrons, and computation of electron‐induced DNA damage.^117^ Lai et al. presented those two new features added to gMicroMC and showed the computation efficiency advantage over the CPU‐based MC computation codes.^117^ The gMicroMC provides us a faster Monte Carlo code sequence, which could make the simulation of biological consequences of different radical's interactions. Those simulations might be even more important than simulation of possible interaction itself.

### 4.2 Discussion

To date, the simulation studies on FLASH still focus on the dosimetry calculation and hardware design. The simulation work on investigating the FLASH effect mechanism is still needed. With FLASH, the radiation progress happens in several microseconds or even shorter in few nanoseconds (pulse mode). The traditional MC simulation tools can present the CONV‐RT progress but are not capable of reproducing the radiolysis in FLASH irradiation. Some newly developed MC toolkits can present the radiolysis of water beyond microsecond^122^ and can investigate the production and evolution of ROS generated with FLASH irradiation. The radiolysis and radiobiology effects in FLASH irradiation are still not fully understood. Many more MC simulation tools need to be developed, especially the ones that can reproduce the progress happening in a short time period of the FLASH irradiation.

## 5 EXPERIMENT

The UHDR irradiation experimental study using electron beams can be traced back to the 1960s, and the first UHDR irradiation experiment was conducted on bacteria in 1959.^14^ More recently, the normal tissue sparing effect under FLASH irradiation was rediscovered and named by Favaudon's group.^7^ Nowadays, more scientists in the community devoted to FLASH research, and a lot more experiment studies were published in the last 5 years. Those experiments could be categorized and discussed in two groups: the in vitro and in vivo experiments. The in vivo experiments were divided into three groups according to their focus: the normal tissue sparing effect under FLASH‐RT, tumor control function of FLASH‐RT, and human patient treatment. In detail, discussion about entire experiment is not performed due to the complexity and diversity of such work. A brief discussion is presented in the final section.

### 5.1 In vitro experiments

The collected studies of in vitro experiments that investigate FLASH effect and mechanism hypothesis are summarized in Table 5. The first FLASH‐related in vitro experiment was performed in 1959, the bacteria irradiation experiment mentioned in the previous section.^14^ Auer et al. performed FLASH and CONV irradiation comparison experiment of cells in 2011.^81^ The G2 HeLa cell monolayer was delivered 3‐Gy dose at CONV and FLASH dose rate. Endpoints were investigated following G2 phase cell cycle arrest, apoptosis, and colony formation. The fraction of G2 cells at FLASH‐RT group was significantly lower than CONV‐RT group 10‐h postirradiation, but no significant difference was observed in other end points. They found out that the RBE of FLASH (pulse) and CONV irradiation were equivalent. Laschinsky et al. conducted an in vitro experiment, in which a traditional accelerator (ELBE) delivered the FLASH electron beam.^68^ They evaluated the difference in response of the normal tissue cell RBE to two delivery modalities (FLASH and CONV), and they confirmed that the RBE of FLASH electron beams was not affected by its UHDR. Buonanno et al. published their investigation of the biological effects in normal cells induced by FLASH‐RT.^41^ They studied the long‐term biological effects of normal human lung fibroblasts exposed to photon and proton irradiation with FLASH and CONV dose rate, and several endpoints were assessed in their work. They found that FLASH‐RT affected the number of senescence cells and expression of TGF‐β1. They concluded that proton FLASH‐RT can reduce delayed detrimental effects. The biological effect of FLASH‐RT on normal cell still needs further investigation, so more study on related in vitro experiments should be performed to make a framework for future clinical work.

### 5.2 In vivo experiments

#### 5.2.1 Normal tissue sparing effect experiments

The first normal tissue sparing effect under UHDR irradiation was reported in 1966.^124^ They found out that mice have a greater survival under FLASH‐RT than those irradiated with conventional dose rate. Favaudon et al. irradiated the C57BL/6J mice with FLASH or CONV dose rate in single doses. The experiment results demonstrated that CONV‐RT on mice triggered lung fibrosis associated with the activation of the TGF‐β cascade after 15‐Gy dose as early as 8 weeks and getting worse by 36 weeks, whereas no complications developed by FLASH‐RT below 20‐Gy dose for over 36 weeks postirradiation. FLASH‐RT also shows the sparing effect on normal smooth and epithelial cells from radiation‐induced apoptosis, comparing to CONV‐RT.^7^ More scientists studied the FLASH effect through irradiation in vivo experiments after Favaudon and Vozenin revealed this novel change in radiation therapy. The recent publications related to in vivo experiments on FLASH effect are shown in Table 6. Montay‐Gruel et al. performed a FLASH irradiation experiment in 2014 where they irradiated the entire brain of the mice at conventional dose rate (0.1 Gy/s) and UHDR (>100 Gy/s). The prototype electron beam LINACs irradiated the mouse's whole brain: Oriatron 6e was used for FLASH‐RT and Kinetron was used for the CONV‐RT. The delivered dose was 10 Gy that was measured with TLD (thermoluminescent dosimeter) chips and other dosimeters. The cognitive tests were performed in this work, which was evaluated by “Novel Object Recognition test” 2‐month postirradiation. Results showed a preservation of mice memory under FLASH‐RT (above 100 Gy/s) 2‐month postirradiation, whereas 10‐Gy WBI CONV‐RT totally impaired the memory. This study showed the FLASH effect on mice’ normal brain tissues for the first time.^54^ Montay‐Gruel et al. published another in vivo experiment study in 2018 and proved the FLASH effect on mice normal brain tissues again. This work provided the first proof that the FLASH effect can be triggered by X‐rays. In that study, the FLASH‐RT was realized with a synchrotron X‐rays source called ESRF, and the CONV‐RT was realized with an XRAD 225Cx (Pxi Precision X‐ray). Twenty‐nine female C57BI/6 mice were used in that work, and 10 Gy was delivered to the entire brain. The cognitive tests were performed and evaluated by “Novel Object Recognition test” 2‐ and 6‐month postirradiation. The results showed that FLASH‐RT WBI does not induce memory deficit, and it reduces hippocampal cell‐division impairment and less reactive astrogliosis.^73^ Simmons et al. provided another evidence of FLASH effect on normal mice’ brain tissues in their in vivo experiment study.^64^ They performed a 30‐Gy WBI to C57BL6/J mice with FLASH dose rate and CONV dose rate. The cognitive tests were conducted by evaluating the spatial and nonspatial object recognition with novel object location and object recognition testing after 10‐week postirradiation. They observed the reduced cognitive impairment and associated neurodegeneration with FLASH‐RT compared with CONV‐RT. Alaghband et al. confirmed the FLASH effect on normal juvenile mice brain tissues in their study.^57^ They found that the FLASH‐RT ameliorates radiation‐induced cognitive dysfunction in multiple independent behavioral paradigms, preserves developing neurons, and limits the reduction of the plasmatic level of growth hormone, compared with CONV‐RT. The first evidence that FLASH‐RT preserves microvasculature integrity in the brain was revealed by Allen et al.^125^ They irradiated the C57BI/6J female mice's whole brain with a single dose of 25 and 10 Gy with CONV and FLASH dose rates. FLASH‐RT was found to reduce levels of apoptosis in the brain at 1‐week postirradiation and to minimize the effect that induced vascular dilation at 1‐week and 1‐month postirradiation. The FLASH‐effect on mice normal brain tissues has been confirmed by Montay‐Gruel's group in several studies.^25^, ^70^, ^126^ The contrary evidence of FLASH effect on mice brain normal tissues is also revealed.^74^ They conducted the TBI (total body irradiation), PBI (abdominal partial body irradiation), and head PBI on C57BLJ/6 mice, using CONV dose rate and FLASH dose rate (37–41 Gy/s). A dose‐escalation study was performed after irradiation, and no evidence of a normal tissue sparing effect was found. Montay‐Gruel et al. proved that the normal tissue–sparing effect occurs under higher irradiation dose rate (above 100 Gy/s).^54^ Ruan et al. found that the higher average dose rate, the larger the FLASH effect in a recently published experiment study on FLASH‐IR in mouse gastrointestinal system.^127^

Vozenin et al. used pig skin to investigate the difference in normal tissue toxicity under FLASH‐RT and CONV‐RT.^99^ A female mini‐pig's skin was delivered a dose range from 22 to 34 Gy with a CONV dose rate (0.01 Gy/s) and a FLASH dose rate (300 Gy/s). The skin response was monitored weekly postirradiation until 48 weeks, and 36 week postirradiation visualization is shown in their paper. The results show that the FLASH‐RT can reduce pig‐skin toxicity. Soto et al. presented an in vivo experiment that shows that FLASH‐RT can reduce mice skin toxicity.^128^ They first revealed the FLASH effect on normal mice skin. Female C57BL/6 mice were delivered 30 and 40 Gy with CONV dose rate and FLASH dose rate (instantaneous pulse dose rate). They scored the skin toxicity according to the depigmentation area size and followed the survival rate postirradiation. The results in their study show that FLASH‐RT can reduce the mice skin toxicity and increase the mice survival rate postirradiation. Abel et al. confirmed that the FLASH‐RT can reduce mice skin toxicity compared to CONV‐RT in their work.^85^ Recently, Cunningham et al. presented a study that the FLASH proton beam can minimize the mice skin toxicity.^87^ They used proton PBS delivery system to irradiate mice at CONV dose rate (1 Gy/s) and two different FLASH dose rates (60 and 115 Gy/s). The plasma and skin levels of TGF‐β, as well as skin toxicity were monitored postirradiation, the results of which show that FLASH‐RT can protect the skin and normal soft tissue. Loo et al. modified a clinical LINAC to perform FLASH‐RT.^129^ Abdomen irradiations of 10 and 22 Gy were delivered to male C57BL/6 mice with CONV and FLASH dose rates. Postirradiation, mice were monitored for survival. The result showed that FLASH‐RT significantly increased survival rate postirradiation, confirming results dating back to the 1960s.^124^ The FLASH‐RT produces less mice mortality, and this is confirmed by Levy et al. in their recent publication.^130^ Venkatesulu et al. performed an in vivo experiment to investigate FLASH effect on normal tissues.^39^ They irradiated the C57BL/6 mice two kinds of dose rates, 0.01 and 35 Gy/s at various delivered dose ranging from 2 to 8 Gy. They assessed the lymphocyte sparing potential in cardiac and splenic irradiation models of lymphopenia and the severity of radiation‐induced gastrointestinal toxicity. The results of that work showed that dose rates of 35 Gy/s do not protect mice from the detrimental side effects of irradiation, contradicting to the reports of others who used the same dose rates (30–100 Gy/s). Moreover, that study also suggested that the FLASH effect on normal tissues might not be universal and additional, yet unknown, biological factors or treatment parameters may impact the FLASH effect. Beyreuther et al. performed the feasibility of proton beam FLASH effect on the normal tissue of zebrafish embryo.^84^ They established a FLASH proton beam delivery system at the University Proton Therapy Dresden. Zebrafish embryos were delivered dose ranging from 0 to 45 Gy at CONV dose rate (0.08 Gy/s) and FLASH dose rate (100 Gy/s). The zebrafish embryo survival rate postirradiation was followed, and the rate of spinal curvature was analyzed in this work. They did not observe a significant difference in embryo survival rate under FLASH‐RT and CONV‐RT. There was only dose point showing the significant difference in the rate of pericardial edema induced by FLASH‐RT and CONV‐RT. The study indicated that more investigation needs to be done on the limitations and requirements of the FLASH effect. Pawelke et al. presented their work recently, which further investigated the FLASH effect of proton beam on zebrafish embryos. They considered the partial oxygen pressure as a relevant parameter.^36^ They irradiated the Zebrafish embryos with 26 Gy dose at CONV and FLASH dose rates. The partial oxygen pressure groups were defined on the basis of the oxygen depletion hypothesis in sealed embryo samples. They observed FLASH effect for most endpoints, ranging from 4% less reduction in embryo to about 20%–25% fewer embryos with spinal curvature and pericardial edema. This work proved that zebrafish embryo is appropriate for FLASH research, and the partial oxygen pressure might be an important parameter in such a study. Velalopoulou et al. presented their in vivo experiment research recently in which they irradiated the mice skin with a proton beam at FLASH and CONV dose rate.^131^ They found that the FLASH‐RT has the equivalent function in control of two murine sarcoma models, but it can reduce toxicity of skin and mesenchymal tissues. A summary of the FLASH effect‐relevant experiments is listed in Table 6.

#### 5.2.2 Experiments on tumor control

The FLASH effect on normal tissues has been observed in many experimental studies, and the radiation oncology community still has a strong interest in investigating whether FLASH‐RT has equivalent tumor control function with CONV‐RT. Favaudon et al. presented a tumor control study under FLASH‐RT in the first FLASH effect publication.^7^ They have also shown that the FLASH‐RT is as effective as CONV‐RT in tumor control through monitoring the growth of human HBCx‐12A and HEp‐2 tumor xenografts and syngeneic TC‐1 LuC+ orthotopic lung tumors in C57BL/6J mice. Zlobinskaya et al. presented a tumor growth control study in 2014.^83^ A laser‐driven ion accelerator was deployed in that work to deliver FLASH dose rate proton beam, in which a clinical LINAC was used to deliver photon reference irradiation. Female NMRI mice, 7–10‐week old, were inoculated in hind legs with exponentially growing FaDu cells. The mice were delivered a dose range from 0 to 40 Gy by photon and proton beam, at CONV and FLASH dose rate, respectively. The tumor size was monitored twice per week postirradiation with diagnostic ultrasound. The results were consistent with other FLASH‐RT tumor control experiments and showed that the FLASH (pulse) proton beam has the similar RBE effectiveness with CONV proton beams. The study also compared the tumor response to proton and photon beams, and the result showed that the proton beams were more effective in tumor growth control. Vozenin et al. presented their FLASH‐RT experiment on cat‐cancer patients in 2019.^99^ Six untreated cats with histologically confirmed SCC (squamous‐cell carcinoma) of the nasal planum, non‐eligible for surgery, were delivered FLASH electron beam irradiation, which was generated by Oriatron 6e. Each of six cat patients received a single pulse range from 25 to 41 Gy. All cats revealed permanent depilation at 18‐month postirradiation. This work confirmed the potential advantage of FLASH‐RT. Diffenderfer et al. presented their design implantation and in vivo experiment work of FLASH‐RT.^79^ C57BL/6J mice were injected with $5\;\times \;10^{5}$ MH641905 cells derived from the KPC autochthonous PanCa model to generate flank tumors. The mice received 12 or 18 Gy with FLASH or CONV dose rate, and tumors were measured with calipers three or four times per week postirradiation. The intestinal fibrosis was evaluated by surgical tumor resection when tumor volume reached 400 mm^3^. FLASH‐RT and CONV‐RT presented a similar dose‐dependent tumor growth control after 12 and 18 Gy, which confirmed that FLASH‐RT as well as CONV‐RT performs to inhibit tumor growth. Cunningham et al. performed the FLASH‐RT with a proton beam and PBS method.^87^ The C57bl/6 mice were injected with MOC1 or MOC2 cells to generate tumors, and the mice were categorized into three groups according to tumor size after 3 weeks. Both delivery modalities showed a great function in tumor growth delay, compared with sham animals. No significant difference was observed between FLASH‐RT and CONV‐RT at each time data point. This work confirmed that the FLASH‐RT with PBS has an equivalent function on tumor growth control with CONV‐RT. Montay‐Gruel et al. published their recent study on FLASH‐RT.^70^ In this work, they investigated the antitumor efficacy and neuroprotective benefits of FLASH‐RT 1‐month postirradiation. The nude mice were injected with H454 orthotropic murine glioblastoma model, after which they received a single dose of 25 Gy with FLASH and CONV dose rate. Tumor development was assessed by contrast‐enhanced cone beam CT before irradiation to provide an accurate visualization of tumors, and these bulkier tumors imaging work was performed by a small animal X‐ray; the tumor volume was measured. FLASH‐RT and CONV‐RT showed a similar function in tumor growth inhibition significantly, compared with Sham animals. Both delivery modalities showed similar neurocognitive functions compared with unirradiated animals. This work also verified the FLASH effect on normal tissues by showing that the FLASH‐RT has the capability of sparing the mouse's normal brain and controlling tumor growth. This exciting FLASH‐RT capability provided the community a framework for future clinical studies. Several in vivo experiments confirmed that FLASH‐RT has the ability to inhibit tumor growth, which are summarized in Table 7.

### 5.3 Clinical trial

The first patient treatment with FLASH‐RT was performed at the Lausanne University Hospital.^56^ The patient was a 75‐year‐old male presented with a CD30^+^ T‐cell cutaneous lymphoma disseminated throughout his skin surface, diagnosed in 1999, and classified as T3 N0 M0 B0. He has received a range of chemotherapeutics treatments since 2001, but none of those treatments could control the disease. He was given local skin RT either with KV X‐rays, low‐energy electrons, or MV X‐rays since 2008, which could, to a degree, control the lymphoma. He was given total of 110 Gy prior to FLASH‐RT treatment. The lymphoma was controlled, but the surrounding skin received too much toxicity. For the FLASH‐RT treatment, 15 Gy was delivered to the 3.5‐cm tumor with a dose rate higher than 10^6^ Gy/s (pulse mode, ≥10^6^ Gy/s, 1.5 Gy/pulse) in 90 ms. The tumor started shrinking around 10‐day postirradiation, whereas the complete tumor response started 36‐day postirradiation, and it lasted for 5 months. A redness was observed in surrounding skin between 10‐ and 44‐day postirradiation, in which asymptomatic mild epithelitis and grade 1 edema were observed between 12‐ and 24‐day postirradiation. The skin reactions did not exceed grade 1, which were smaller and disappeared in a much shorter time compared with the patient's previous CONV‐RT. The first FLASH‐RT clinical treatment was feasible and safe, confirming that the FLASH‐RT can protect the human normal skin and control the tumor.

A canine cancer patient with a large oral malignant melanoma in the caudal part of the hard palate was treated with FLASH‐RT by a modified clinical LINAC. The patient received two treatments of 35 Gy each with an average dose rate at 280 Gy/s, which resulted in prolonged survival and better quality‐of‐life.^72^, ^132^ The United States has started a clinical study trial called Feasibility Study of FLASH RT for the Treatment of Symptomatic Bone Metastases (FAST‐01), the study designed to assess the workflow feasibility of FLASH‐RT in patient treatment setting and also the toxicities and pain relief to treat bone metastasis. CHUV (Centre hospitalier universitaire vaudois), the hospital where treated the first human patient with FLASH‐RT has announced that they started treatment of multiple skin cancer patients within a clinical trial.

## 6 SUMMARY AND CHALLENGES

FLASH‐RT appears to be a revolutionary tumor treatment modality and gets the attention of many scientists in the radiation oncology community. We presented this review to summarize the history and status of FLASH‐RT studies as well as to pinpoint the existing challenges and prospects of this novel technique. Successfully delivered FLASH irradiation publications are listed and divided into three categories (electron, photon, and proton) according to the radiation source type. The current hypotheses to explain the FLASH effect's underlying mechanism are discussed in this manuscript with related theoretical and experimental work mentioned. Most of the in vivo and in vitro experiments are summarized and discussed in this paper to the best of our knowledge. MC simulations are broadly used in medical physics community and their applications in FLASH‐RT are listed in this manuscript.

Although many exciting developments have been made in FLASH‐RT recently, there are many obstacles that need to be overcome to translate FLASH into clinic. The FLASH‐RT is still not ready for human treatment as several techniques need to be developed. There is still no such delivery system that can deliver multiple (usually five to seven) FLASH irradiation beams simultaneously, which is required state‐of‐the‐art in CONV‐RT. The CONV‐RT intensity modulation needs to be upgraded into a sub‐second scale to suit FLASH‐RT. The current real‐time adaptation needs to be improved to ensure that the beam and target are aligned under FLASH irradiation. The unclear underlying mechanism of FLASH effect is another challenge in applying FLASH‐RT into clinic. Several mechanisms studies have been presented recently with some fundamental radiobiological processes hypothesized and understood. Regardless, a deeper understanding of FLASH‐RT‐related radiolysis and cellular processes is required before a clinical application. It is important to understand the difference between biological changes induced by FLASH and CONV irradiation. To date, studies have presented the early effect of radiation, but the late and overall effects of FLASH‐RT are still unknown. Eventually, FLASH‐RT will be translated into clinical application scientifically, rather than phenomenologically. The scientific findings about FLASH effect still need to be verified independently. The FLASH versus CONV studies with the dose rates as the only controlled variable need to be performed in the future. Even though MC simulations can help scientists understand the FLASH‐RT, to date, there is no MC simulation platform that can reproduce the FLASH irradiation process, from the radiation transport to radiolysis in tissues and DNA damage at subcellular level. The advanced MC simulation tools need to be developed to speed up the FLASH effect's underlying mechanism. Many questions remain regarding the mechanism and clinical feasibility of FLASH‐RT, and this will be the future major goal of radiation oncology community to decrypt the code of FLASH effect.

## AUTHOR CONTRIBUTION

*Yuan Gao*: conceptualization, methodology, investigation, writing—original draft, visualization. *Ruirui Liu*: methodology, writing—review and editing. *Chih‐Wei Chang*: methodology, writing—review and editing. *Serdar Charyyev*: writing—review and editing. *Jun Zhou*: writing—review and editing. *Jeffrey D. Bradley*: writing—review, editing, supervision. *Tian Liu*: writing—review and editing, supervision. *Xiaofeng Yang*: conceptualization, writing—review and editing, supervision, project administration, and funding acquisition.

## CONFLICT OF INTEREST

The authors declare that there is no conflict of interest that could be perceived as prejudicing the impartiality of the research reported.
